# The impact of COVID-19 and associated measures on health, police, and non-government organisation service utilisation related to violence against women and children

**DOI:** 10.1186/s12889-022-12644-9

**Published:** 2022-02-12

**Authors:** Nadia Butler, Zara Quigg, Isabelle Pearson, Zhamin Yelgezekova, Aasa Nihlén, Mark A. Bellis, Yongjie Yon, Jonathon Passmore, Isabel Yordi Aguirre, Heidi Stöckl

**Affiliations:** 1 Public Health Institute, Liverpool John Moores University, Liverpool, UK; 2 Gender Violence and Health Centre, Department of Global Health and Development, London School of Hygiene and Tropical Medicine, London, UK; 3 World Health Organization, Geneva, Switzerland; 4 College of Health and Behavioural Sciences, Bangor University, Bangor, UK; 5 Policy and International Health Directorate, Public Health Wales, Clwydian House, Wrexham, UK; 6 Institute for Medical Information Processing, Biometry, and Epidemiology, Pettenkofer School of Public Health, Ludwig-Maximilians-Universität München, Munich, Germany

**Keywords:** COVID-19, Violence against women, Violence against children, Service provision

## Abstract

**Background:**

Globally, concerns have been raised that the priority implementation of public health measures in response to COVID-19 may have unintended negative impacts on a variety of other health and wellbeing factors, including violence. This study examined the impact of COVID-19 response measures on changes in violence against women and children (VAWC) service utilisation across European countries.

**Methods:**

A rapid assessment design was used to compile data including a survey distributed across WHO Europe Healthy Cities Networks and Violence Injury Prevention Focal Points in WHO European Region member states, and a scoping review of media reports, journal articles, and reports. Searches were conducted in English and Russian and covered the period between 1 January 2020 and 17 September 2020. Data extracted included: country; violence type; service sector; and change in service utilisation during COVID-19. All data pertained to the period during which COVID-19 related public health measures were implemented compared to a period before restrictions were in place.

**Results:**

Overall, findings suggested that there was a median reported increase in VAWC service utilisation of approximately 20% during the COVID-19 pandemic. Crucially, however, change in service utilisation differed across sectors. After categorising each estimate as reflecting an increase or decrease in VAWC service utilisation, there was a significant association between sector and change in service utilisation; the majority of NGO estimates (95.1%) showed an increase in utilisation, compared to 58.2% of law enforcement estimates and 42.9% of health and social care estimates.

**Conclusions:**

The variation across sectors in changes in VAWC service utilisation has important implications for policymakers in the event of ongoing and future restrictions related to COVID-19, and more generally during other times of prolonged presence in the home. The increased global attention on VAWC during the pandemic should be used to drive forward the agenda on prevention, increase access to services, and implement better data collection mechanisms to ensure the momentum and increased focus on VAWC during the pandemic is not wasted.

**Supplementary Information:**

The online version contains supplementary material available at 10.1186/s12889-022-12644-9.

## Background

The novel severe acute respiratory syndrome coronavirus 2 (SARS-CoV-2), which causes the COVID-19 disease, was identified in Wuhan, China in December 2019, and by March 2020 had spread to over 100 countries and been declared a global pandemic by the World Health Organization (WHO) [1]. At the time, with no population immunity, available vaccine, or effective treatment for COVID-19, countries implemented various public health policies to limit exposure and manage population risk. Amongst measures such as contact tracing, physical distancing, face masks, and hygiene protocols, more stringent restrictions were put in place by many European countries from March 2020 including school and workplace closures, bans on public gatherings, and stay-at-home requirements [2].

Whilst such measures were intended to reduce the spread of COVID-19, concerns were raised about the potential for restrictions on movements to negatively impact other aspects of health and wellbeing. A particular area of concern was the impact of the restrictions on levels of violence and crucially, the ability of organisations to prevent and respond to violence. Whilst violence in public spaces was predicted to decrease as a result of stay-at-home orders and the closure of night-time economies, non-governmental organisations (NGOs) and other stakeholders working in the field of violence prevention warned of the risk of a ‘shadow pandemic’, where such measures compounded existing violence and abuse in private settings, such as the home, and exacerbated risk factors for already vulnerable individuals, particularly women and children [3–7].

Globally, prior to the pandemic, over 1 billion children experience sexual, physical, or psychological abuse each year, whilst one in three women will experience intimate partner violence and/or sexual assault in their lifetime [8, 9]. Some evidence from previous epidemics suggests violence against women and children (VAWC) may increase during the COVID-19 pandemic [10, 11]. During the Ebola virus outbreaks in the Democratic Republic of Congo and Sierra Leone, qualitative studies showed children experienced more frequent physical violence and community members perceived an increase in violence against women and girls [12, 13]. A global systematic review of the impact of natural disasters on violence amongst women and girls (VAWG), found a positive association between disaster exposure and increases in VAWG with qualitative findings suggesting such increases may be a result of stressors that emerge in post-disaster settings (e.g. economic insecurity), enabling environments (e.g. reduced police activity), and exacerbation of underlying drivers of VAWG (e.g. gender inequalities) [14]. Similarly, COVID-19 and the associated responses have exacerbated many of the risk factors associated with VAWC including health and economic stresses, cramped living conditions, parental stress and tension, children’s increased presence in the home, isolation with perpetrators, and deserted public spaces [3, 5, 7, 15]. Increases in these stressors may not only increase the risk of victimisation for the first time but also increase the frequency or severity of violence for those already experiencing violence and abuse [16, 17]. Crucially, the increased risk of violence is compounded by greater difficulties accessing support services and informal sources of support such as family and friends [18, 19]. Movement restrictions and social distancing measures, as well as reduction and reallocation of resources in victim-related services, decrease opportunities to identify victims of violence in health, education, and employment settings, reduce victims’ ability to access services, and limit the support services can provide [3, 5, 15]. A systematic review of child abuse during natural disasters and conflicts found that while levels of violence against children increased during such periods, reporting of violence decreased due to disruptions in services, infrastructure, and reporting mechanisms [16].

Without population-level surveys it is difficult to obtain current rates of VAWC across countries, and even more difficult to assess how levels of VAWC may have changed during the pandemic [15]. Whilst there are known limitations with administrative data including underreporting, underrepresentation of particular groups, and lack of comparability across countries [20], service-based or administrative data can provide valuable insight into the number of women and children using such services. It can also demonstrate the need for such services and estimate the resources required to maintain them [20] and, in the case of the COVID-19 pandemic, understand how the direct infectious disease response measures affect service access and use. Understanding the status of violence prevention and response services during the COVID-19 pandemic is thus crucial for mapping gaps in availability and accessibility and for the future allocation of resources to ensure continued support for victims of violence during future restrictions. The current study aimed to identify administrative service level data across the 53 WHO European Member States to examine whether COVID-19 response measures impacted VAWC service use during the early stages of the pandemic.

## Methods

### Study design

Due to the time-sensitive nature of identifying the initial impact of COVID-19 on VAWC, the current study used a rapid assessment design to compile data on changes in service provision across WHO European Region. The rapid assessment used both primary and secondary data collection methods including a survey distributed across WHO European Healthy Cities Networks (EHCN; [21]) and Violence Injury Prevention Focal Points (VIPFP; [22]) in the WHO European Region [23], and a scoping review of media reports, journal articles, and NGO and government reports.

### Survey

The survey was distributed via the WHO Regional Office for Europe on behalf of the research team to individuals who are official focal points for different strands of WHO work, representing either: co-ordinators from a city member of the WHO EHCN; and/or WHO technical focal points for violence and injury prevention (nominated by Ministries of Health). The survey was available in English, Italian, and Russian, and asked participants to provide information and data on the impact of COVID-19 on service use related to VAWC. Informed consent was collected from all participants. Thirty-seven survey responses were received from individuals in the following member states: Austria, Azerbaijan, Bosnia and Herzegovina, Croatia, Czechia, Denmark, Finland, France, Georgia, Greece, Iceland, Italy, Luxembourg, Montenegro, North Macedonia, Norway, Portugal, the Russian Federation, Serbia, Spain, and the United Kingdom.

### Scoping review

Searches were conducted in English and Russian and covered the period between 1 January and 17 September 2020. Searches were conducted by two researchers (one for the English language searches and one for Russian language searches), with results exported into Excel and duplicates removed. English language searches were conducted in the International Newsstream of ProQuest (limited to the Asian, European, and Middle Eastern Newsstreams), OVID (restricted to OVID MEDLINE®, and In-Process & Other Non-Indexed Citations), and Google Scholar. Russian language searches were conducted in East View Information Service, Yandex.ru, Radio Liberty’s Central Asian branches, Sputniknews, and Google Scholar (including eLibrary.ru and CyberLenika). Grey literature searches of relevant national and international organisational websites were also conducted in English and Russian. All references retrieved were subjected to an include/exclude process based on the full text. The inclusion criteria were that the publication contained data: related to VAWC service utilisation during the COVID-19 pandemic; related to one or more of the 53 Member States of the WHO European Region; and, suitable for extraction and synthesis (i.e. quantitative data in the form of percentage change or raw data with which percentage change could be calculated). Three reviewers screened the full text to identify literature for inclusion, with 10% of data extracted by each reviewer (excluding data from the Russian language search) checked by a second reviewer.

### Measures

For each source, data was extracted on: country; income level of the country (using World Bank categorisation); violence type; service sector (health and social services; law enforcement; or NGOs); change in service utilisation during COVID-19; and, comparison period. In some instances, the same data was reported in more than one source. In such cases, only one change in service utilisation figure was extracted to reduce duplicate estimates, and all other extracted information was combined to increase the completeness of data. All service data were included regardless of the measure it related to (e.g. incidents, reports, crimes, calls, online chats). Website visit data was excluded as an increase in visits to websites does not necessarily reflect an increase in utilisation of the service. Service utilisation data were extracted as percentage change or calculated from raw data. All data pertained to the period during which COVID-19 related public health measures were implemented compared to a period before restrictions were in place and were country-specific. Percentage change was calculated by either comparing to the same period in 2019 (e.g. March 2020 vs. March 2019) or the same period before lockdown in 2020 (e.g. March 2020 vs. February 2020). In some cases, the comparison period used to calculate the percentage change was not specified.

### Statistical analysis

Data were analysed using SPSS (v.25). Analyses employed descriptive statistics to calculate frequencies and medians. Chi-square for independence was used for bivariate examination of associations between service sector and change in service utilisation (binary variable coding each estimate as increase or decrease in utilisation). To ensure the comparison period used to calculate the percentage change did not confound any relationship between change in service utilisation and sector, logistic regression was employed to examine the independent relationship between service sector and change in service utilisation, while controlling for the comparison period (including not specified). Modelled estimates for proportion of data points categorised as an increase in service demand were calculated for each sector using an estimated marginal means function to adjust estimates for comparison period [24]. Sensitivity analysis was also undertaken using a definition of increase or decrease in service utilisation as a change ≥  ± 20 percentage points to reduce the risk of small meaningful variations contributing to statistical differences.

## Results

Overall, 350 data points related to VAWC service utilisation during our COVID-19 study period were identified from the survey and scoping review. Additional file 1 (Table A1) provides the number of data points extracted by sector, country and country income level, violence type, and comparison period.

**Fig. 1:**
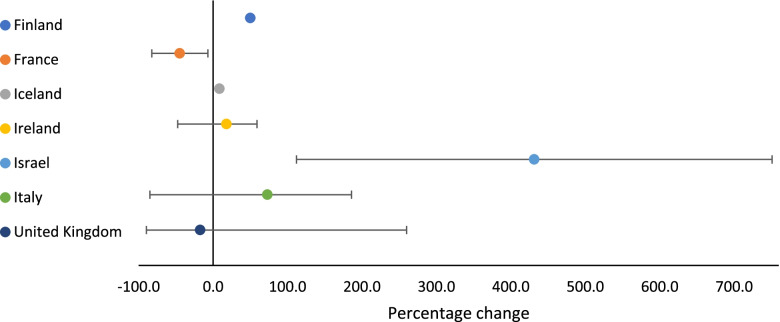
Percentage change in health and social service utilisation related to VAWC during COVID-19, by country (median, minimum and maximum values)

**Fig. 2:**
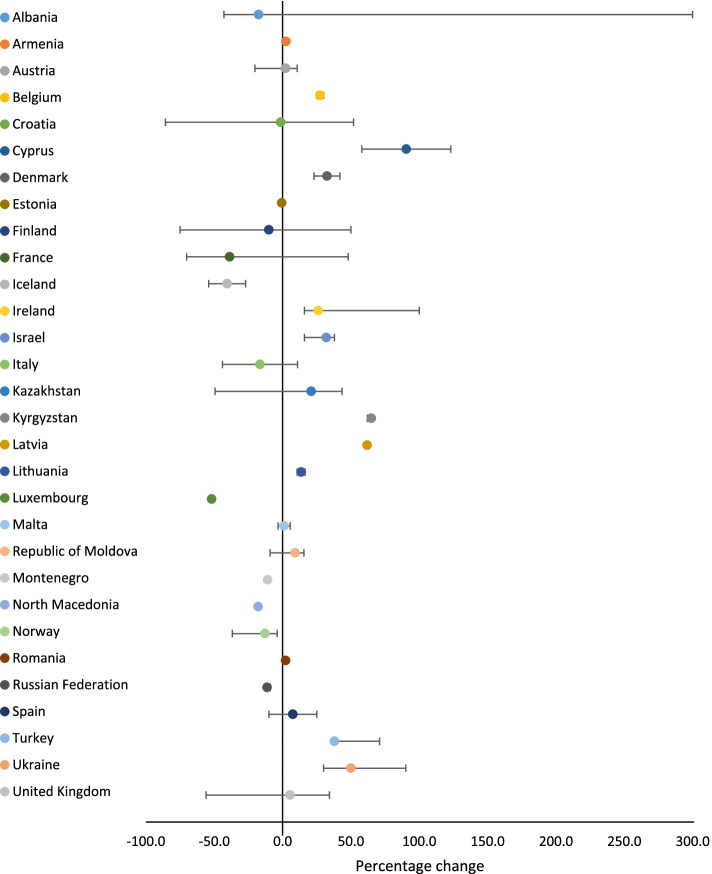
Percentage change in law enforcement service utilisation related to VAWC during COVID-19, by country (median, minimum and maximum values)

**Fig. 3:**
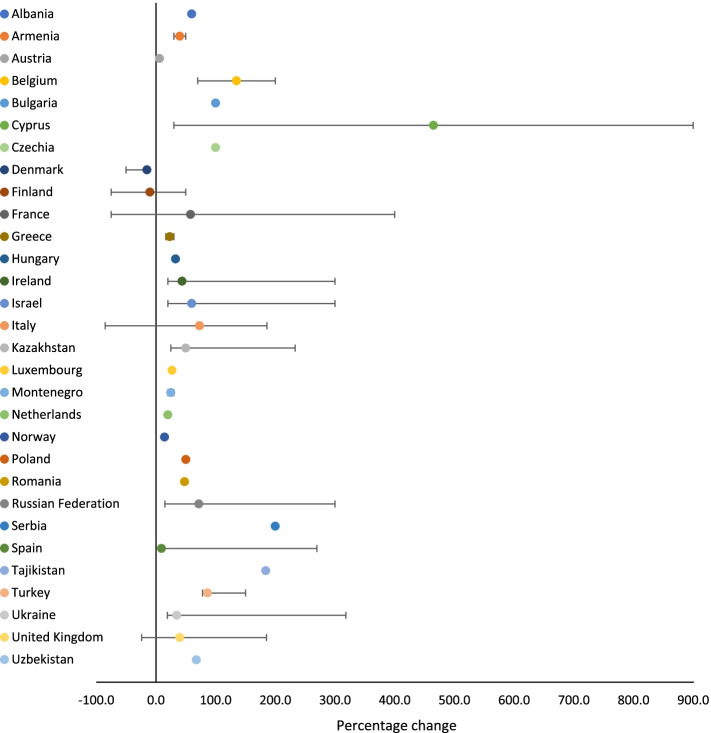
Percentage change in NGO service utilisation related to VAWC during COVID-19, by country (median, minimum and maximum values)

Overall, across all three sectors, there was a 20.3% median increase in service utilisation (range: -9.6%—900.0%), with a median increase in service utilisation of 5.8% (range: -85.7%—300.0%) for law enforcement, and 47.5% (range: -85.0%—900.0%) for NGOs. Use of health and social services saw a median decrease of 8.0% (range: -89.6%—751.0%). The median percentage change in service utilisation across countries for each sector is presented in Figs. 1, 2 and 3.

**Fig. 4:**
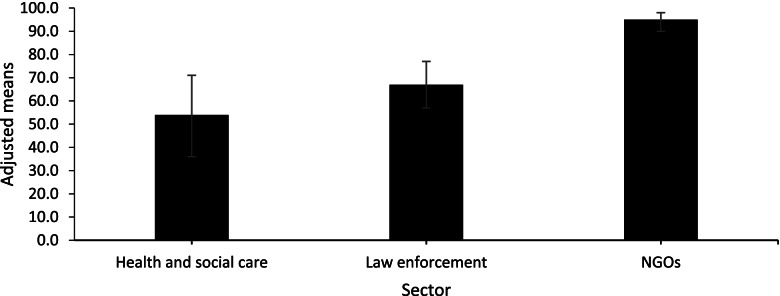
Adjusted proportion (95% confidence interval) of data points categorised as an increase in VAWC service utilisation during COVID-19, by service sector

Each percentage change data point was then categorised as an increase or decrease in VAWC service utilisation during COVID-19. Bivariate analyses showed a significant association between sector and changes in service utilisation (χ^2^ = 69.76; *p* < 0.001). Most NGO estimates (95.1%; *n* = 135) reflected an increase in service utilisation, compared to 58.2% (*n* = 96) of law enforcement estimates, and 42.9% (*n* = 18) of health and social care estimates. In the multivariate analysis, after controlling for the type of comparison period used to calculate the percentage change in service utilisation during COVID-19, health and social service data was 17.23 (95% CI: 6.16–48.18) times as likely to show a decrease compared to NGO data, whilst law enforcement data was 9.69 (95% CI: 4.10–22.94) times as likely to show a decrease (Table 1). Modelled estimates reflect these findings (Fig. 4), with the highest proportion of data points reflecting an increase in service utilisation related to NGO data (95.0%), followed by law enforcement data (67.0%), and health and social care data (54.0%). Sensitivity analysis using ≥  ± 20 as cut-offs to categorise each estimate as a significant increase or decrease (or no change) in service utilisation had no significant impact on the significance or interpretation of the results (see Additional file 1: Tables A2 and A3).

**Table 1:** Binary logistic regression analysis of relationships between change in service utilisation, sector, and comparison period

	**Decrease in service utilisation (reference category: increase)**		
	AOR	95% CI	*p*
**Sector**			
NGOs (ref.)			
Health and social care	17.23	6.16–48.18	< 0.001
Law enforcement	9.69	4.09–22.94	< 0.001
**Comparison period**			
Not specified (ref.)			
Same period pre lockdown	2.11	0.66–6.80	0.211
Same period previous year	4.12	1.93–8.77	< 0.001

## Discussion

In the early stages of the COVID-19 pandemic, there was global concern from stakeholders working in the field of violence prevention that, as a result of the public health restrictions in place to limit the spread of the virus, levels of violence would rise and violence support services would be inaccessible [3–6]. Without large-scale population-level surveys, accurately determining changes in incidence or prevalence of violence during the early stages of the pandemic was not feasible [20, 25, 26]. However, administrative data from health and social services, law enforcement, and NGOs were more readily available and provided an initial understanding of levels of service utilisation. The current study used emerging administrative service data from the early stage of the COVID-19 pandemic to examine changes in service utilisation related to VAWC. Overall, findings suggested that there was a median increase in service utilisation related to VAWC of approximately 20% during the early stages of the pandemic. Crucially, however, change in service utilisation differed across sectors with the highest median increase in utilisation for NGOs (48%), a relatively small median increase for law enforcement services (6%), and a small decrease for health and social services (-8%). After categorising each estimate as reflecting an increase or decrease in VAWC service utilisation during the pandemic, there was a significant association between sector and change in service utilisation, with most NGO estimates (95.1%) showing an increase in utilisation, compared to 58.2% of law enforcement estimates, and 42.9% of health and social care estimates. After controlling for the comparison period used to calculate the change in utilisation, multivariate analysis showed health and social service data was 17 times more likely to show a decrease compared to NGO data, whilst law enforcement data was almost 10 times as likely to show a decrease.

Findings in the current study are in line with other global evidence which demonstrates that COVID-19 related disruption of VAWC services is not equally distributed across service types and sectors [15, 17, 27]. A study by UNICEF reported that whilst 66% of countries surveyed reported overall disruptions to VAC-related services, this differed by service type, with only 12% of countries reporting disruptions to child protection helplines compared to 48% reporting disruptions to child welfare authorities [17]. Reports from civil society, women’s rights organizations, and humanitarian services have reported increases in calls and service utilisation related to VAW since the beginning of the COVID-19 pandemic [27]. However, evidence suggests disruptions to law enforcement and health services have been more varied, and whilst increases in utilisation were reported in some countries, many reported decreases in reports of VAW [15]. Thus, evidence suggests that NGO’s, particularly in terms of helpline calls, saw increases in service utilisation during COVID-19, however, use of health services and law enforcement related to VAWC was more varied across countries, and in many cases suggested a decrease in utilisation during the early stages of the pandemic [15, 17, 27].

There are several possible reasons why VAWC service utilisation may have varied across sectors during the pandemic. Without population-level surveys it is difficult to assess any true changes in levels of violence during the pandemic, however, some emerging evidence indicates increased severity of violence and resulting injuries [28]. Thus the fluctuations across and even within sectors in the current study are more likely to represent victims’ ability and willingness to access services during lockdown, rather than actual changes in levels of violence [25]. Existing data prior to the pandemic suggest the majority of cases of violence against women never come to the attention of the authorities. Global self-reported survey data shows that whilst 31% of women aged 15–49 years have experienced physical and/or sexual violence in their lifetime [29], only 40% of women who experience violence report it to someone, and of those who do less than 10% report the incident to the police [30]. Similarly, global evidence suggests self-reported child sexual abuse is more than thirty times higher, and physical abuse more than 75 times higher, than official reports or cases detected by child welfare authorities [31, 32]. A safe environment and a trusted relationship is often a necessary condition for disclosure of violence or abuse [33, 34]. NGOs with established trusted relationships with service users prior to COVID-19 may have thus been better equipped to support clients during the pandemic, than services such as police and health where such relationships are less likely. Further, NGOs in several European countries reported proactively making contact with known survivors and those at risk of violence or abuse to ensure they were safe and to offer support [35].

Those experiencing violence may also have been unwilling to attend health care services during the pandemic for fear of contracting the virus or placing additional burden on the health care system, with emergency departments across Europe showing decreases in attendance related to a range of health concerns during lockdown [36–38]. Furthermore, in the initial stages of lockdown, confusion around stay-at-home orders may have meant victims were unaware they could still seek medical or police assistance. This was addressed in many countries in later months with the implementation of communication campaigns that aimed to increase reporting and highlight stay-at-home orders did not apply to those fleeing abusive homes [35]. Even in cases where survivors presented at health care services, time and space to safely screen for, and identify abuse may have been more difficult. A key response measure in many countries was the reallocation of resources, particularly with respect to health services and law enforcement, to the direct infectious disease response, and thus resource for other activities such as violence prevention and response were reduced, particularly multi-sector coordinated responses [3]. Mandated sources of reporting of child abuse such as teachers, child care providers, and clinicians had fewer opportunities to detect and report signs of abuse during the pandemic due to reduced in-person provision [39–41]. Whilst some countries introduced legislation to help statutory services adapt to remote working and facilitate stay-at-home orders, in some cases this added further barriers to providing victims with support [40, 41]. For example, in the United Kingdom, temporary amendments to children’s social care regulations came into place in April 2020 to no longer require a social worker to perform home visits to a child in care every six weeks, reducing it to ‘as soon as is reasonably practicable’, whilst in France consultation in child welfare services were restricted to emergencies and parent–child meetings in the presence of a social worker were suspended [40, 42]. Thus, services that were traditionally more reliant on in-person provision, such as health services and safeguarding personnel (e.g. teachers), were heavily restricted in their ability to identify victims and provide support. Conversely, many NGOs already had online or telephone service provision in place prior to the pandemic, and thus were better equipped to provide remote support. Furthermore, it is possible that the initial reluctance to attend health services or seek police assistance during the early stages of the pandemic may have led to some of the increase in utilisation of NGO support services.

The findings from the current study, and other early studies on COVID-19 and VAWC, have several important implications and considerations for policymakers in the event of ongoing and future measures related to COVID-19 that limit service provision and access. Crucially, these considerations also have relevance to violence prevention work in future emergencies, natural disasters, and more generally for other times of prolonged presence in the home, for example during Christmas and summer holidays [16]. Firstly, the pandemic has highlighted the crucial role NGOs play in violence prevention and providing support for victims. NGO victim support services must therefore be included on the list of designated essential services that are allowed to continue delivery in the case of any future restrictions [43]. Furthermore, with predictions of a post-COVID-19 global recession [44], and associated cuts to service funding likely, governments should ensure funding is allocated to this sector, to scale up and adapt services where necessary. Lessons on how to engage with survivors and the importance of a trusting relationship and safe space to disclose violence can also be utilised by other sectors. The emerging model of trauma-informed services in many countries is an important step in realising this and providing stakeholders across multiple sectors with the knowledge and skills to initiate disclosures and signpost survivors to appropriate support [45].

Secondly, the pandemic forced many services to quickly adapt to remote working, and to integrate new technologies and innovative services into their offer to ensure provision to their service users was as uninterrupted as possible [41, 46]. The necessity of the situation forced many services to substantially upgrade IT systems in a matter of weeks which otherwise may have taken months or years, in addition to making increased use of technologies such as video conferencing platforms to replace face-to-face meetings [41, 46]. This enabled services to use new and innovative ways of reaching victims and providing them with access to support. For example, in Poland, a fictitious online cosmetic store was set up on Facebook where women experiencing violence could request help by pretending to order products [47]. The French organisation L’Enfant Bleu used a gaming platform to provide a communication route for children and young people experiencing abuse to report it and access support [47]. Such adaptations have the potential to increase service accessibility to victims, particularly where the perpetrator is present in the home and accessing helplines is more difficult, however further research is needed to assess if this approach is effective [48]. Consideration also needs to be given to the potential for a digital divide; many victims from low-and-middle-income countries do not have access to online technology or phone services, with an estimated 445 million ‘unconnected’ adult women globally, thus face-to-face provision remains crucial [49].

Thirdly, whilst the pandemic has brought increased global attention to VAWC, it has also highlighted the difficulties measuring the prevalence, nature, and impact of VAWC, particularly in emergencies such as COVID-19. For example, the current study was limited by having to primarily rely on media-reported changes in levels of service utilisation during the initial stages of the pandemic. Further, even attempts to directly contact country’s key stakeholders in violence prevention to request data was limited, with many unable to provide data, and even where data was provided, problems remained with data quality (e.g. lack of pre-pandemic comparative data). Good data is critical to informing evidence-based policies and tailored programmes to respond to the needs of women and children during and after the pandemic, as well as during any future public health emergencies, conflicts, or natural disasters [50]. For example, in Wales, United Kingdom, a pre-existing violence surveillance system that collates datasets from multiple sectors including police, health, and NGOs, was used to monitor the impact of COVID-19 on levels of violence, and inform targeted responses, prevention activity, and communication and awareness campaigns. [4]. Crucially, data was also used to highlight the extent of violence in private settings and prevent existing violence prevention resources from being reallocated from sectors such as public health and police to the direct infectious disease response [47]. Administrative data systems are often better equipped to measure violence in public spaces than in the home where the victims are more likely to be women and children. Since violence prevention resource allocation is often determined by data evidencing where there is need, creation and consideration of datasets (e.g. NGOs) is crucial. They can provide an indication of levels of more hidden forms of violence both during the current pandemic and in violence prevention efforts more generally. The integration of such datasets in national surveillance systems is recommended best practice by WHO in the Global Action Plan to strengthen the role of the health system, within a multisector response to address VAWC [51]. Such approaches should be used to supplement the information collected in population-based surveys which are the best method for determining the prevalence of VAWC (but still represent an underestimate) and include standardised measures and indicators allowing comparability across countries and regions [20].

Findings in the current study should be interpreted in light of a number of considerations. Comparison periods used to calculate the percentage change in service utilisation varied across the collected estimates. However, we tried to mitigate the potential confounding of this factor by performing a multivariate analysis controlling for comparison period. Whilst data is presented and grouped based on the data source (e.g. police recorded data), comparisons should not be drawn between different countries. Differences between countries in, for example, legal definitions, recording practices, and data quality, mean figures are not directly comparable. Further, data may be related to different time periods or measures (e.g. police data may include incidents, reports, or crimes). As already discussed, much of the estimates were drawn from media sources which may introduce bias as these sources may be more likely to report ‘interesting’ or ‘shocking’ statistics and we had no way to check the accuracy of the reports. Whilst the study focused on VAWC service utilisation across the whole WHO European Region our searches were conducted in English and Russian and thus may have missed media reports in other languages. Further, whilst the survey requesting data from key stakeholders was sent to representatives across the whole WHO European Region, the quality of responses varied greatly.

## Conclusions

The COVID-19 pandemic may eventually wane, however, the impacts of the public health restrictions on other aspects of health and wellbeing may continue to affect individuals, communities, and societies in the long term. Findings from the current study suggest that during the pandemic, even fewer victims were reporting violence to the police or presenting at health services. However, NGO data suggested that violence had not reduced amongst these groups during lockdown, and service utilisation overall increased. National and international campaigns highlighting the impact of COVID-19 on the increased risk of VAWC in the home captured the attention of the global media and galvanised governments into action in many countries [47]. Governments, public authorities, and stakeholders working in the field of violence prevention should use the increased focus on VAWC during the pandemic to drive forward the agenda on prevention, implement better data collection mechanisms, and crucially increase access to services both during periods of global crises and ordinary times. Public expenditure cuts are likely given the predicted recession, but global evidence suggests that investment in early life services is cost-effective in reducing violence against children [52], which is critical because violence against children is linked to violence revictimisation across the life course [53]. The COVID-19 pandemic has put the spotlight on VAWC – some of the most hidden and underreported forms of interpersonal violence. With the reopening of society, and in particular, night-time economies and the reappearance of violence in public spaces, the momentum and focus on violence in the home during the pandemic should not be wasted.

## Disclaimer

The authors alone are responsible for the views expressed in this publication and they do not necessarily represent the decisions or policies of the World Health Organization.

## Supplementary Information

**Additional file 1. **

## Data Availability

The dataset generated and analysed in the current study is available from the corresponding author on reasonable request.

## Abbreviations

AOR: Adjusted odds ratio
CI: Confidence interval
EHCN: European Healthy Cities Networks
NGO: Non-government organisation
WHO: World Health Organization
VAC: Violence against children
VAW: Violence against women
VAWC: Violence against women and children
VAWG: Violence against women and girls
VIPFP: Violence Injury Prevention Focal Points
