# Supplementary material for: The impact of COVID-19 and associated measures on health, police, and non-government organisation service utilisation related to violence against women and children
Source: BMC Public Health. 2022 Feb 12;22:288. doi: 10.1186/s12889-022-12644-9 (PMC8840226; doi:10.1186/s12889-022-12644-9)
Supplement: Supplementary file 1 — Additional file 1. [file 12889_2022_12644_MOESM1_ESM.docx]

**ADDITIONAL FILE 1**

**Table 1: Data points extracted by sector, country, country income level, violence type and comparison period**

|  | N | % |
| --- | --- | --- |
| Sector |  |  |
| Health and social services | 43 | 12.3 |
| Law enforcement | 165 | 47.1 |
| NGOs | 142 | 40.6 |
| Country income level |  |  |
| High income | 269 | 76.9 |
| Upper-middle income | 52 | 14.9 |
| Lower-middle income | 29 | 8.3 |
| Country |  |  |
| Albania | 9 | 2.6 |
| Armenia | 3 | 0.9 |
| Austria | 5 | 1.4 |
| Belgium | 4 | 1.1 |
| Bulgaria | 1 | 0.3 |
| Croatia | 9 | 2.6 |
| Cyprus | 4 | 1.1 |
| Czechia | 1 | 0.3 |
| Denmark | 5 | 1.4 |
| Estonia | 1 | 0.3 |
| Finland | 4 | 1.1 |
| France | 28 | 8.0 |
| Greece | 2 | 0.6 |
| Hungary | 1 | 0.3 |
| Iceland | 4 | 1.1 |
| Ireland | 21 | 6.0 |
| Israel | 17 | 4.9 |
| Italy | 11 | 3.1 |
| Kazakhstan | 11 | 3.1 |
| Kyrgyzstan | 3 | 0.9 |
| Latvia | 1 | 0.3 |
| Lithuania | 2 | 0.6 |
| Luxemburg | 2 | 0.6 |
| Malta | 2 | 0.6 |
| Moldova | 5 | 1.4 |
| Montenegro | 3 | 0.9 |
| Netherlands | 1 | 0.3 |
| North Macedonia | 1 | 0.3 |
| Norway | 7 | 2.0 |
| Poland | 1 | 0.3 |
| Romania | 2 | 0.6 |
| The Russian Federation | 16 | 4.6 |
| Serbia | 2 | 0.6 |
| Spain | 13 | 3.7 |
| Tajikistan | 1 | 0.3 |
| Turkey | 7 | 2.0 |
| Ukraine | 16 | 4.6 |
| UK | 123 | 35.1 |
| Uzbekistan | 1 | 0.3 |
| Violence type |  |  |
| Violence against women | 283 | 81.8 |
| Violence against children | 63 | 18.2 |
| Comparison period |  |  |
| Same period pre lockdown | 51 | 55.7 |
| Same period previous year | 195 | 14.6 |
| Not specified | 104 | 29.7 |

**Table 2: Bivariate sensitivity analysis of association between sector and change in service utilisation**

|  | Change in service demand | | | ꭓ^2^ | p |
| --- | --- | --- | --- | --- | --- |
| Sector | **Decrease** | **No change** | **Increase** |  |  |
|  | **% (n)** | **% (n)** | **% (n)** |  |  |
| Health and social services | 41.9% (18) | 27.9% (12) | 30.2% (13) |  |  |
| Law enforcement | 23.0% (38) | 44.8% (74) | 32.1% (53) |  |  |
| NGOs | 3.5% (5) | 12.7% (18) | 83.8% (119) | 103.409 | <0.001 |

**Table 3: Multinomial regression sensitivity analysis of relationships between change in service utilisation, sector and comparison period**

|  | Service utilisation (reference category: no change) | | | | | | |
| --- | --- | --- | --- | --- | --- | --- | --- |
|  | **Decrease** |  |  |  | **Increase** |  |  |
|  | **AOR** | **95% CI** | **P** |  | **AOR** | **95% CI** | **p** |
| Sector |  |  |  |  |  |  |  |
| NGOs (ref.) |  |  |  |  |  |  |  |
| Health and social care | 4.33 | 1.21-15.45 | <0.05 |  | 0.25 | 0.09-0.68 | <0.01 |
| Law enforcement | 1.53 | 0.51-4.6 | 0.450 |  | 0.14 | 0.07-0.28 | <0.001 |
| Comparison period |  |  |  |  |  |  |  |
| Not know (ref.) |  |  |  |  |  |  |  |
| Same period previous year | 0.34 | 0.05-2.1 | 0.243 |  | 0.22 | 0.08-0.60 | <0.01 |
| Same period pre lockdown | 0.95 | 0.32-2.82 | 0.93 |  | 0.12 | 0.05-0.25 | <0.001 |
